# Intravenous fluids: balancing solutions

**DOI:** 10.1007/s40620-016-0363-9

**Published:** 2016-11-29

**Authors:** Ewout J. Hoorn

**Affiliations:** Division of Nephrology and Transplantation, Department of Internal Medicine, Erasmus Medical Center, Room D-438, PO Box 2040, 3000 CA Rotterdam, The Netherlands

**Keywords:** Balanced crystalloids, Hyponatremia, Hypotonic fluids, Hyperchloremic acidosis, Kidney transplantation, Pediatrics

## Abstract

The topic of intravenous (IV) fluids may be regarded as “reverse nephrology”, because nephrologists usually treat to remove fluids rather than to infuse them. However, because nephrology is deeply rooted in fluid, electrolyte, and acid-base balance, IV fluids belong in the realm of our specialty. The field of IV fluid therapy is in motion due to the increasing use of balanced crystalloids, partly fueled by the advent of new solutions. This review aims to capture these recent developments by critically evaluating the current evidence base. It will review both indications and complications of IV fluid therapy, including the characteristics of the currently available solutions. It will also cover the use of IV fluids in specific settings such as kidney transplantation and pediatrics. Finally, this review will address the pathogenesis of saline-induced hyperchloremic acidosis, its potential effect on outcomes, and the question if this should lead to a definitive switch to balanced solutions.

## Introduction

In a sense, the topic of intravenous fluids is “reverse nephrology”. Nephrologists usually spend their days removing fluids, either by diuretics or by ultrafiltration, rather than infusing them. Still, nephrologists like to think of themselves as experts in fluid, electrolyte, and acid-base balance. This is directly linked to IV fluids as IV fluid therapy implies infusing fluid, electrolytes, and buffers directly into the extracellular fluid volume. IV fluids can be used to correct electrolyte or acid-base disorders, while—equally so—their inadequate use can cause these disorders (Tables 1, 2). Nephrologists are often asked for advice on IV fluids in consultative services, for example in the intensive care, surgical or medical wards. Regardless of the context, the proper prescription of IV fluids requires understanding of physiology. Or, stated more eloquently, “their appropriate use requires reverence for the fine balance that constitutes human homeostasis [1].” A useful approach is to consider IV fluids as drugs, including specific pharmacokinetic and pharmacodynamics properties (as recently reviewed by us [2]). In addition, to understand the current selection of the types of IV fluids, it is useful to briefly review the history of IV fluids in medicine. One of the pioneers in applying IV fluids was Dr. Thomas Latta who, during the cholera epidemic in 1832, instead of infusing fluid into the colon, decided to “throw it immediately in the circulation” [3]. He used a fluid consisting of “soda and two scruples of subcarbonate of soda in six pints of water”, which was remarkably effective, but also quickly forgotten [4]. Between 1882 and 1885 the British physiologist Dr. Sydney Ringer conducted his so-called “extravital investigations” and developed a fluid which enabled a frog’s heart to continue beating outside the body by using a solution comparable to blood plasma [5, 6]. In 1932 the American pediatrician Dr. Alexis Hartmann modified Ringer’s solution by adding the buffer lactate [7, 8]. The IV fluid lactated Ringer’s or Hartmann’s solution still reminds us today of these achievements and may be considered the first balanced solutions (Table 3). Around 1900 the Dutch physiologist Dr. Hartog Jacob Hamburger developed “physiological” or “normal” saline, i.e. 0.9% sodium chloride [9, 10]. In retrospect, the adjectives “physiological” and “normal” may be considered misnomers, because especially the chloride concentration in 0.9% NaCl is supraphysiological (154 vs. 100 mmol/l, Table 3) [11]. The fluids developed by Latta, Ringer, Hartmann, and Hamburger all qualify as *crystalloids*, water with electrolytes forming a solution. Crystalloids should be distinguished from *colloids*, in which—chemically speaking—insoluble particles are suspended, but not in solution. A naturally occurring colloid is albumin, which was first used to treat trauma casualties, including burn patients after the Pearl Harbor attack [12]. Today, albumin is occasionally used in severe hypovolemic shock, and in patients with liver failure [13]. Other colloids include dextrans, hydroxyethyl starches, and gelatins. Colloids will not be reviewed in detail here, because they are being used less frequently after several signals that they may cause harm (increased mortality [14] and acute kidney injury (AKI) [15]), especially after retraction of the articles by Dr. Joachim Boldt [16]. Instead, this review will focus on the evolving field of crystalloid IV fluids.

**Table 1:** Indications for intravenous fluids

Replace extracellular fluid volume losses
Maintain fluid and electrolyte balance
Correct existing electrolyte or acid-base disorders
Provide a source of glucose

**Table 2:** Complications of intravenous fluids

Typical type of IV fluid	Complication
Normal saline	Hyperchloremic metabolic acidosis, worsening hypertension
Hypotonic IV fluids	Hyponatremia
Hypertonic or isotonic IV fluids	Hypernatremia
Usually isotonic IV fluids	Fluid overload

**Table 3:** Composition of commonly used intravenous fluids

	Osmolality	Tonicity	Na^+^	Cl^−^	K^+^	Mg^2+^	Ca^2+^	Buffer^a^
Plasma	288	Reference	140	103	4.5	1.25	2.5	24
0.9% NaCl	308	Isotonic	154	154	0	0	0	0
Lactated Ringer’s	279	Hypotonic	130	111	4.0	0	2.7	29
PlasmaLyte	N/A	Isotonic	140	98	5.0	1.5	0	50
Sterofundin	309	Isotonic	140	127	4.0	1.0	2.5	29
5% Glucose	278	Hypotonic	0	0	0	0	0	0
1.4% NaHCO_3_	333	Hypertonic	167	0	0	0	0	167

All in mmol/l, except for osmolality in mOsm/kg. *N/A* not available

^a^Buffers consist of bicarbonate (plasma, NaHCO_3_), lactate (lactated Ringer’s), acetate (27 mmol/l in PlasmaLyte, 24 mmol/l in Sterofundin), gluconate (23 mmol/l in PlasmaLyte), and maleate (5 mmol/l in Sterofundin)

## Indications for intravenous fluids

IV fluids are so ubiquitous in clinical medicine that one would almost forget considering its indications (Table 1). An important classification is the distinction between replacement and maintenance IV fluids. Patients requiring replacement IV fluids have a degree of volume depletion that may be due to hemorrhagic or non-hemorrhagic causes. For both categories, the rapid infusion of isotonic saline is indicated for resuscitation (e.g., 500 ml in 10 min, repeated as needed). Isotonic IV fluids expand the intravascular compartment more effectively than hypotonic IV fluids. No studies are available to address whether balanced crystalloids offer advantages in this setting [17]. Colloids are not recommended because of their adverse effects (discussed above) and also because they may be less effective [18]. In less severe volume depletion, the goal of replacement therapy is to correct existing abnormalities in fluid, electrolyte, or acid-base balance. Furthermore, IV fluids may be used in patients with pre-renal azotemia in the context of AKI or acute on chronic renal insufficiency. The fractional excretion of urea rather than sodium may be useful to select patients eligible for a trial of fluid repletion [19]. Maintenance IV fluids are usually given when the patient cannot drink for a prolonged period of time. Owing to immobility, hospitalized patients often require less than a liter of water per day. However, this rough estimate can change dramatically in circumstances with increased loss due to fever, sweating, burns, tachypnea, gastro-intestinal losses, drains, or polyuria. Conversely, non-osmotic stimuli may be present for the release of vasopressin (the antidiuretic hormone), resulting in renal water retention [20]. Changes in body weight and the serum sodium concentration (as measure of water balance) are useful parameters to assess water balance and, accordingly, plan initial IV fluid therapy. Other parameters should also guide the selection of IV fluid therapy, including blood pressure, acid-base status, kidney function, and the presence of diabetes. In general, isotonic IV fluids are recommended for maintenance [20], but specific settings may require tailored therapy. Recommended average rates of infusion are 100–120 ml/h, but should be decreased (25 ml/h in oligoanuric states, 40–60 ml/h in edematous states) or increased (>120 ml/h with urinary concentrating defect) depending on the clinical context [20]. The tendency towards isotonic maintenance IV fluids may be related to previous cases of acute hyponatremia, for example due to the combination of post-operative vasopressin release and the use of hypotonic IV fluids [21]. In addition, because IV fluid therapy can cause fluid overload, and a positive fluid balance in the ICU is associated with higher mortality [22], the need for giving maintenance fluids should always be critically reviewed [23]. Although IV fluids often contain glucose, it offers a poor source of long-term nutrition, which should usually come from enteral tube feeding. Alternatively, in patients with compromised gut function, total parental nutrition may be indicated. These considerations raise the question if glucose should be part of maintenance IV fluids. This practice differs per country and no large studies are available comparing isotonic maintenance fluids with or without dextrose. One randomized trial in the peri-operative setting did show that 72% of patients receiving IV fluids containing dextrose developed transient hyperglycemia, whereas those without dextrose remained normoglycemic [24]. Because hyperglycemia is associated with worse outcomes after acute neurological injury, dextrose may need to be avoided especially in this setting [25, 26]. When hypoglycemia is a potential risk (e.g., during surgery in children) dextrose levels can be safely reduced from 5 to 1% (Table 4) [27].

**Table 4:** Answered and unanswered questions

Sufficient evidence	Unanswered questions
Crystalloids have fewer side-effects than colloids	Are balanced crystalloids better than normal saline?
Normal saline can cause hyperchloremic acidosis and impair coagulation	Which balanced crystalloid is preferable?
Isotonic rather than hypotonic maintenance IV fluids are preferable in pediatrics	Should maintenance IV fluids contain glucose?

## Etiology of saline-induced acidosis

Normal saline has long been the dominant type of IV fluid both for replacement and maintenance. A unique side-effect of normal saline is hyperchloremic metabolic acidosis. A straightforward question without a clear answer is why “normal” saline causes hyperchloremic acidosis. Normal saline differs from other IV fluids in two regards: it does not contain a buffer and it has a higher chloride concentration. Indeed, both the change in serum chloride and the volume of infused 0.9% NaCl correlated with the degree of acidosis in one study [28]. One possibility, therefore, is that expansion of the extracellular fluid with a buffer-free fluid dilutes serum bicarbonate and therefore causes acidosis. This phenomenon of “dilution acidosis”, however, is not linear because experimental data showed that a 28% expansion of the extracellular fluid volume reduced serum bicarbonate by only 10% [29]. The degree of acidosis is likely attenuated by mobilization of intracellular bicarbonate (e.g., from bone) and binding of hydrogen ions by albumin and hemoglobin. Subsequent clinical cases, however, did find the degree of acidosis to be predictable by the amount of normal saline [30]. Both Gattioni et al. and Doberer et al. performed in vitro experiments to investigate the basis of dilution acidosis [31, 32]. They concluded that, for dilution acidosis to occur, the infused volume should largely exceed urine output, and an open CO_2_/HCO_3_^−^ buffer system should exist, where the buffer base (HCO_3_^−^) but not the buffer acid (CO_2_) is diluted [31]. In a commentary accompanying both studies, Davenport offered yet another explanation focusing on the observation that hyperchloremic acidosis usually occurs in a phase of clinical improvement [33]. He suggested that improved organ perfusion results in a “wash out” of lactate, organic acids, and other intermediary metabolites. This, however, should increase the serum anion gap, which is usually normal in hyperchloremic acidosis. Dilution acidosis is also unlikely to be a renal phenomenon (i.e., due to reduced bicarbonate reabsorption or reduced proton excretion), because it has also been reported in patients with end-stage renal disease receiving normal saline [34], and the kidney responds with an appropriate increase in NH4^+^ excretion [35].

## Comparing solutions

The development of hyperchloremic metabolic acidosis during IV fluid therapy has led to the search for alternative options. Continuous hemorrhage experiments in dogs showed that resuscitation with lactated Ringer’s kept blood pH stable at 7.40, while normal saline reduced it slightly to 7.36 [36]. In humans, a 2-h peri-operative infusion of 30 ml/kg/h normal saline but not lactated Ringer’s increased serum chloride (104 to 115 mmol/l) and reduced pH (7.40 to 7.28) [37]. The authors considered this phenomenon benign unless confused with hypoperfusion or when superimposed with respiratory acidosis by analgetics in the post-operative phase. In a double-blind randomized trial, patients who received normal saline while undergoing aortic reconstructive surgery required more bicarbonate supplementation and more blood products [38]. However, no differences in the duration of mechanical ventilation, intensive care or hospital stay, and incidence of complications were noted [38]. Subsequent studies confirmed the differential effects on coagulation of IV fluids, ascribing this both to a dilutional coagulopathy by normal saline [39] and the development of hypercoagulability with lactated Ringer’s [40]. Together, these head-to-head comparisons clearly show that normal saline causes hyperchloremic metabolic acidosis and impairs coagulation. Because these fluids were given for relatively short periods of time (peri-operatively or during resuscitation after trauma), long-term outcomes were more difficult to evaluate. Still, these studies likely spurred the development of newer balanced crystalloid solutions, including PlasmaLyte and Sterofundin (Table 3). For PlasmaLyte and Sterofundin other buffers than lactate are used (acetate and maleate) and these solutions are isotonic compared to the mildly hypotonic lactated Ringer’s [41]. Since their introduction, several studies have been performed with these newer balanced crystalloids. In healthy volunteers, 2 l of normal saline but not PlasmaLyte increased serum chloride and reduced renal artery flow velocity and renal cortical tissue perfusion (as measured by magnetic resonance imaging) [42]. This effect may be a direct consequence of hyperchloremia, which has been shown to produce renal vasoconstriction and a fall in glomerular filtration rate [43]. This effect on renal blood flow may have contributed to a greater expansion of the extracellular fluid volume with normal saline. Indeed, the excretion of both water and sodium are slower after 2 l of normal saline than after a balanced solution [11]. Because the hypertensive effect of sodium also depends on chloride, normal saline may increase blood pressure, especially in hypertensive patients [44]. Although observational, a study on postoperative IV fluids also favored PlasmaLyte over normal saline [45]. The use of normal saline after open abdominal surgery was associated with higher mortality, and more infections, blood transfusions, renal replacement therapy (RRT), electrolyte disorders, and acidosis [45]. One of the first large prospective studies was an open-label, sequential period pilot study comparing a so-called “chloride-liberal” IV fluid regimen (normal saline, gelatin, albumin) to a “chloride-restrictive” regimen (Hartmann’s solution, PlasmaLyte, chloride-poor albumin) [46]. The chloride-restrictive regimen nearly halved the risk of AKI and RRT. In a 6-month extension of this study these differences in renal outcomes remained [47]. In contrast, the more recent and controlled SPLIT trial (double-blind and double-crossover) compared normal saline with PlasmaLyte in 2278 ICU-patients and found no differences in the incidences of AKI or RRT [48]. Of note, this study did not report serum chloride concentrations and a relatively small volume (median of 2 l) was infused in patients with moderate severity of disease [49]. Finally, and quite surprisingly, no sample size calculations were performed [48].

## Intravenous fluids in specific settings

### Kidney transplantation

If high-chloride IV fluids indeed impair renal blood flow or even kidney function, the type of IV fluid during kidney transplantation may be particularly relevant. O’Malley et al. addressed this issue in a double-blind randomized trial comparing lactated Ringer’s to normal saline (primary outcome was serum creatinine at day 3) [50]. The study was terminated prematurely because significantly more patients in the normal saline group developed hyperchloremic acidosis and hyperkalemia (serum potassium >6 mmol/l) [50]. Of interest, urine output and graft function were also significantly worse in the normal saline group even a few days after transplantation. Recently, a Cochrane analysis was performed on this topic including the six studies that have been performed to date (477 patients, 70% live donor) [51]. In this analysis, the use of normal saline was associated with a higher risk of hyperchloremic acidosis, but not hyperkalemia or delayed graft function. No intervention studies in other solid organ transplantations are available. However, an observational study in 158 liver transplantations identified the use of >3.2 l of chloride-liberal fluids and a higher pre-operative model for end-stage liver disease (MELD) score as independent predictors for postoperative AKI (occurring in a third of the patients) [52].

### Pediatrics

Until recently IV fluids in pediatrics were usually hypotonic. Maintenance requirements for water were traditionally based on caloric expenditure [53]. This usually resulted in glucose-containing solutions with less than 0.9% NaCl (e.g., 0.67, 0.45 or 0.2% NaCl). Because glucose is metabolized to carbon dioxide and water, the net result is a hypotonic solution. This may become problematic if there is a reason for the release of vasopressin. A myriad of non-osmotic stimuli for vasopressin is recognized, including hypovolemia, nausea, pain, the postoperatieve state, several drugs or underlying disease (ranging from infections to malignancy) [20]. Indeed, several cases of iatrogenic acute hyponatremia due to hypotonic IV fluids have been reported in children [54]. In a previous observational study, we showed that almost half of the children with hyponatremia developed this in-hospital while receiving more electrolyte-free water in IV fluids [55]. In children with gastroenteritis plasma levels of vasopressin were frequently high due to vomiting, hypovolemia, hypoglycemia, or “stress” [56]. Consequently, most children had a urinary tonicity that exceeded that of 0.45% saline, predisposing them to the development of hyponatremia. In a randomized trial, the same group subsequently showed that isotonic IV fluids in children with gastroenteritis effectively treat hyponatremia present on admission while preventing hospital-acquired hyponatremia [57]. More rapid infusion of IV fluids, however, did not correct dehydration faster [57]. When considering the pediatric patient population as a whole, a systematic review (including six studies) confirmed that hypotonic solutions greatly increased the risk of developing hyponatremia [58]. One of the first randomized controlled trials that compared 0.45% NaCl with 0.9% NaCl in 5% dextrose as maintenance IV-fluids, however, did not detect an effect on serum sodium, but likely lacked power [59]. A larger trial comparing the same solutions postoperatively did demonstrate a greater risk for hyponatremia with hypotonic fluids [60]. Two meta-analyses, both including ten randomized controlled trials, again confirmed the risk of hyponatremia with hypotonic fluids [61, 62]. Despite this increasing body of evidence, two additional trials were conducted recently [63, 64]. Friedman and colleagues randomized 110 children to receive isotonic (0.9% NaCl in 5% dextrose) or hypotonic IV fluids (0.45% NaCl in 5% dextrose) at maintenance rates for 48 h [63]. The difference in serum sodium concentration after 48 h was minimal (1.4 mmol/l), although the two children developing hyponatremia were both in the hypotonic IV fluid group. The study by McNab et al. had a similar set-up, but is of interest for two reasons [64]. First, the sample size was large: 690 children who required IV fluids for over 6 h. Second, PlasmaLyte was used as isotonic IV fluid and was compared with a hypotonic IV fluid containing 77 mmol/l sodium. Patients in the isotonic group developed hyponatremia significantly less often (11% vs. 4%). Although cerebral edema was not more common in the hypotonic group, a trend towards more seizures was observed (7 vs. 1 children, p = 0.07). In all of these studies isotonic IV fluids did not increase the risk of hypernatremia or fluid overload [65]. Overall, the message is crystal clear: similar to adults, children also require isotonic maintenance IV fluids, and the focus should now be on the implementation of this compelling evidence [66].

## Intravenous fluids to correct electrolyte or acid-base disorders

In addition to replacing fluid loss or maintaining fluid balance, IV fluids may also be used specifically to correct an existing electrolyte or acid-base disorder. IV fluids are instrumental in the treatment of certain subtypes of hyponatremia, hypernatremia, metabolic acidosis, and metabolic alkalosis, which will be discussed here. IV fluids can also be used as vehicle to correct other electrolyte disorders (hypokalemia, hypocalcemia, hypomagnesemia, and hypophosphatemia) but this is beyond the scope of this review. Because hyponatremia always indicates a positive water balance, water restriction or approaches to increase renal water excretion are usually the mainstay of treatment. However, patients with hyponatremia can be truly hypovolemic (e.g., due to vomiting or diarrhea), in which case water balance is less negative (relatively positive) in comparison to sodium balance. In these patients hyponatremia can be corrected by isotonic IV fluids. Because vasopressin release is driven by hypovolemia, a sudden water diuresis may occur when the extracellular volume normalizes during IV fluid therapy with the risk of (too) rapid correction of hyponatremia [67]. In addition, acute or symptomatic hyponatremia is usually treated by specific IV fluids, namely hypertonic saline (usually 3% NaCl). In this context, the tonicity rather than the volume of the IV fluid is relevant, as this hypertonic solution will attract water from the intracellular compartment. As such, hypertonic saline can be used to treat cerebral edema in hyponatremic encephalopathy. The amount of hypertonic saline that should be administered can be calculated by the Adrogué–Madias formula [68]. More recently, the approach of giving a bolus of hypertonic saline has been advocated because it can be given rapidly in emergency situations while avoiding calculations [69, 70]. However, a strong evidence base for these recommendations is lacking. Ayus et al. did recently report their experience with a protocol of uniformly infusing 500 ml 3% hypertonic saline in 6 h in 64 patients with hyponatremic encephalopathy [71]. Although this approach appeared safe and effective, the patients had severe hyponatremia (average serum sodium 114 ± 0.8 mmol/l), in whom the distinction between acute or chronic (or acute on chronic) hyponatremia is often difficult to make. In addition, it is important to factor in body weight [72]. In contrast to hyponatremia, hypernatremia should be treated with hypotonic fluids [73]. If possible, increasing oral water intake is preferable, but otherwise half-isotonic or 5% dextrose can be used (while avoiding hyperglycemia). Of note, patients presenting with hypernatremia can be severely hypovolemic and may require initial resuscitation with isotonic saline (that is still relatively hypotonic to serum sodium). In patients in whom volume depletion is accompanied by metabolic acidosis, sodium bicarbonate rather than sodium chloride may be the preferred solution. This strategy may be particularly effective in patients who lost bicarbonate due to gastrointestinal losses. Intravenous sodium bicarbonate is usually reserved for acute metabolic acidosis, but it remains controversial when to initiate treatment (usually when pH <7.1). In addition, the focus should be on the underlying cause of the acidosis (e.g., ketoacids, lactate, or intoxications). If intravenous sodium bicarbonate is given, calculating the bicarbonate space helps to assess the amount of sodium bicarbonate. Commercially available solutions with preset concentrations of sodium bicarbonate may be used (e.g., 1.4, 4.2 or 8.4%, Table 3). Depending on the concentration of sodium bicarbonate used, hypernatremia is a potential side-effect [74]. An alternative solution in this regard is tris-hydroxymethylaminomethase (THAM), an amino alcohol which buffers acids and carbon dioxide [75]. In patients with lactic acidosis due to sepsis or a low-flow state, sodium bicarbonate does not improve outcomes [76, 77]. Experimentally, the negative effects of sodium bicarbonate (lowering of serum calcium and generation of carbon dioxide) can be effectively targeted by calcium supplementation and hyperventilation, but clinical trials are lacking [76, 78]. The much-criticized high chloride concentration of normal saline may actually benefit patients with so-called chloride depletion metabolic alkalosis [79, 80]. This usually concerns patients who lost large amounts of hydrogen chloride from their upper gastrointestinal tract. In these patients hypochloremia is central to the pathogenesis of metabolic alkalosis, as it will directly impair renal bicarbonate excretion. Thus, whatever the place of the newer balanced crystalloid solution may become, normal saline will still remain the treatment of choice for chloride depletion metabolic alkalosis.

## Conclusions

Electrolyte solutions are part of our daily clinical practice. The use of isotonic rather than hypotonic maintenance IV fluids is now well established, especially in pediatrics (Table 4) [64]. The comparison between normal saline and balanced crystalloid solutions, however, is still undecided. Although balanced crystalloid IV fluids have physiological appeal given their resemblance to human plasma (Table 3), strong evidence in favor of their general use is still lacking (Table 4). Normal saline can cause hyperchloremic acidosis, which, in turn, may negatively affect renal blood flow [42, 43]. Although preliminary studies indeed showed a higher degree of AKI and RRT in patients treated with normal saline compared to balanced crystalloids [46, 47], a recent large randomized controlled trial failed to confirm these differences [48]. Such differences in outcome may be especially evident in patients receiving large volumes of IV fluids, providing a potential focus for future trials. Similarly, in the setting of kidney transplantation, prevention of hyperchloremic acidosis may be most relevant in patients receiving a cadaveric donor, in whom delayed graft function is a common problem. Regardless of these interesting developments in the field of IV fluids, the first critical assessment should be whether the patient actually requires IV fluids, and if so, to tailor this to the specific characteristics of that individual patient.
