# Correction to: Intravenous fluids: balancing solutions

**DOI:** 10.1007/s40620-020-00704-5

**Published:** 2020-01-28

**Authors:** Ewout J. Hoorn

**Affiliations:** Division of Nephrology and Transplantation, Department of Internal Medicine, Erasmus Medical Center, Room Ns410, PO Box 2040, 3000 CA Rotterdam, The Netherlands

## Correction to: J Nephrol (2017) 30:485–492 10.1007/s40620-016-0363-9

In Table 3 on Page 486 the term osmolality should have read osmolarity. For an overview of both osmolality and osmolarity for each of the intravenous fluids, please see the following reference:

Severs D, Hoorn EJ, Rookmaaker MB. A critical appraisal of intravenous fluids: from the physiological basis to clinical evidence. Nephrol Dial Transplant. 2015;30(2):178–87.

